# The Impact of Individuals’ Social Environments on Contact Tracing App Use: Survey Study

**DOI:** 10.2196/45825

**Published:** 2023-05-31

**Authors:** Atiyeh Sadeghi, Sebastian Pape, David Harborth

**Affiliations:** 1 Chair of Mobile Business & Multilateral Security Faculty of Economics and Business Goethe University Frankfurt Frankfurt Germany

**Keywords:** contact tracing app, corona warning app, Corona-Warn-App, social influence, usage, COVID-19

## Abstract

**Background:**

The German Corona-Warn-App (CWA) is a contact tracing app to mitigate the spread of SARS-CoV-2. As of today, it has been downloaded approximately 45 million times.

**Objective:**

This study aims to investigate the influence of (non)users’ social environments on the usage of the CWA during 2 periods with relatively lower death rates and higher death rates caused by SARS-CoV-2.

**Methods:**

We conducted a longitudinal survey study in Germany with 833 participants in 2 waves to investigate how participants perceive their peer groups’ opinion about making use of the German CWA to mitigate the risk of SARS-CoV-2. In addition, we asked whether this perceived opinion, in turn, influences the participants with respect to their own decision to use the CWA. We analyzed these questions with generalized estimating equations. Further, 2 related sample tests were performed to test for differences between users of the CWA and nonusers and between the 2 points in time (wave 1 with the highest death rates observable during the pandemic in Germany versus wave 2 with significantly lower death rates).

**Results:**

Participants perceived that peer groups have a positive opinion toward using the CWA, with more positive opinions by the media, family doctors, politicians, and virologists/Robert Koch Institute and a lower, only slightly negative opinion originating from social media. Users of the CWA perceived their peer groups’ opinions about using the app as more positive than nonusers do. Furthermore, the perceived positive opinion of the media (*P*=.001) and politicians (*P*<.001) was significantly lower in wave 2 compared with that in wave 1. The perceived opinion of friends and family (*P*<.001) as well as their perceived influence (*P*=.02) among nonusers toward using the CWA was significantly higher in the latter period compared with that in wave 1. The influence of virologists (in Germany primarily communicated via the Robert Koch Institute) had the highest positive effect on using the CWA (B=0.363, *P*<.001). We only found 1 decreasing effect of the influence of politicians (B=–0.098, *P*=.04).

**Conclusions:**

Opinions of peer groups play an important role when it comes to the adoption of the CWA. Our results show that the influence of virologists/Robert Koch Institute and family/friends exerts the strongest effect on participants’ decisions to use the CWA while politicians had a slightly negative influence. Our results also indicate that it is crucial to accompany the introduction of such a contact tracing app with explanations and a media campaign to support its adoption that is backed up by political decision makers and subject matter experts.

## Introduction

### Background

With the global pandemic caused by SARS-CoV-2, digital proximity tracing systems to identify people who have been in contact with an infected person are one approach to trying to get the pandemic under control. There have been many discussions on different implementations and their architecture [1], that is, whether the approach should be centralized or decentralized. One implementation is the German Corona-Warn-App (CWA). It is built with privacy in mind, is based on a decentralized approach [2], and the usage intention of German citizens has already been widely discussed concerning privacy concerns [3] and knowledge about the app [4]. However, the influence of different groups in the social environments of citizens on the use of contact tracing apps during the pandemic was—to the best of our knowledge—not a subject of extensive research before. This is interesting from a theoretical point of view because research on the acceptance of new technologies considers social influence as an antecedent of behavioral intention to use technologies [5]. Consequently, it also found its way [6,7] into some successors of the Technology Acceptance Model (TAM; cf. [8]).

Furthermore, this lack of research on social influence and contact tracing apps is surprising because the medical nature of the disease (SARS-CoV-2) is inherently based on human interactions. Furthermore, previous research suggests that knowledge about the CWA significantly reduces the privacy concerns about it [4]. However, most citizens do not acquire knowledge from primary sources but rather from discussions with their peer groups. Thus, the assumption must be made that the decision to undertake a disease prevention measure (in our case using a contact tracing app) is always embedded within the back and forth of social interactions, perceptions, or even pressures. This can also be seen in the design of contact tracing apps. They not only allow their users to see whether they had potential contact with infected individuals but also to warn others by entering positive (or negative) SARS-CoV-2 test results. Consequently, it is crucial to investigate how citizens perceive the opinion of their peer groups on using contact tracing apps. However, because this question alone would not suffice to draw conclusions on the decision of the citizens to use the app, we also need to ask whether this opinion influences them for or against using such an app. To address this, we conducted a longitudinal survey study with 833 participants to investigate these opinions and the perceived influence of a set of peer groups on the participants to use the CWA. Peer groups in our study include media (eg, print media, websites, and television), family doctors, politicians, virologists/the Robert Koch Institute (RKI; a German federal government agency and research institute responsible for disease control and prevention), social media, and friends and family.

We surveyed participants 2 times with a time distance between the surveys of approximately 10 months to also investigate changes over time of the use behavior of the app and the opinions and influences of the relevant groups and to control for the severeness of the pandemic. These 2 periods were chosen because we observed the height of the death rates due to SARS-CoV-2 in Germany during the first period (wave 1) with more than 1200 deaths at a given day compared with significantly lower death rates during the second period (wave 2) with approximately 200 deaths at a given day.

In summary, we investigate the following 4 research questions (RQs):

- RQ1: How do users and nonusers perceive opinions of relevant groups and their influence?
- RQ2: What are the differences between users and nonusers?
- RQ3: How do the opinions and the influence change over time (from wave 1 to wave 2) driven by infection rates (decreased from wave 1 to wave 2)?
- RQ4: How does the opinion of the relevant groups influence the usage of the CWA?

### Prior Work

Researchers have conducted surveys on adopting SARS-CoV-2 tracing apps in various countries [9]. Although some data point to reasonably high app support globally [10], other research highlighted the issue of low usage rates [11]. The majority of articles use surveys to investigate the users’ adoption of 1 or more contact tracing apps (eg, in Australia [12], China [13], France [10], Germany [3,4,10,13,14], Ireland [15,16], Italy [10], Taiwan [17], the United Kingdom [10,18,19], and the United States [10,13,20]). For example, Horstmann et al [21] (see also [3]) found for a sample in Germany that the most common reasons for nonusers were privacy concerns, lack of technical equipment, and doubts about the app’s eﬀectiveness. Most other studies reported similar results and identiﬁed privacy concerns as one of the main barriers to using contact tracing apps. In particular, people are worried about corporate or government surveillance, potentially even after the pandemic [16], leakage of data to third parties [10], exposure of social interactions [22], and secondary use of the provided data [22]. However, misconceptions based on widespread knowledge gaps accompany the adoption of contract tracing apps [4].

Besides these studies, Blom et al [23] studied potential adoption barriers of the official contact tracing app (Corona-Warn-App) that was launched in Germany on June 16, 2020.

Their findings indicate that with low adoption rates in the general population and problems with selectivity across subgroups, the data reflect a pessimistic view of the usefulness of app-based contact tracing to contain the SARS-CoV-2 epidemic in Germany. According to their estimates, roughly 81% of the German population aged between 18 and 77 years have access to devices that can be used to install the German Corona-Warn-App. However, the authors found that only 35% are eager to do so. This indicates that most citizens lack awareness about the app or the motivation to use it. Thus, research is needed to investigate individuals’ reasons for and against using the app.

Previous studies have focused on users’ perceptions and motivations concerning mobile health (mHealth) apps on a more general level without considering the aspect of social interactions and pressure, which are associated with a technology focusing on combating infectious diseases [24-26]. According to prior research on individuals’ motivations for using mHealth apps, factors such as access to a smartphone with the necessary app installed and internet connectivity [27,28], smartphone users’ capacity to carry out the functions necessary to use the app [29], prior experience using mobile technologies [30,31], reliable information and true performance and functionality provided by the apps [32-35], trust in data security or authorities [10,14,36,37], and privacy concerns [16,24,38-51] have a significant role in their motivation to use mHealth apps.

Less research has, however, examined the effect of social influence and social relationships [10,14,16,52-59] on the motivation to use mHealth apps, especially in the context of infectious disease presentation (which effectively is the target of contact tracing apps). For mHealth apps in general, research finds that the more people identify with others, the more positively they view these other individuals [60-62]. The degree of identification with the source (or “authority”) predicts the propensity of individuals to utilize these new technologies [63,64]. Social influence is also used in related research, which uses the TAM to investigate factors influencing users’ willingness to use and pay for a mobile health care app [59]. Bettiga et al [59] incorporated the idea of social influence through subjective norms that play a crucial part in decisions and health-related choices. A subjective norm is defined as an individual’s sense of the level to which significant others approve or disapprove of the target behavior [65]. Self-care and preventative behavior are frequently driven by a sense of compliance to social expectations from family members, the social group to which the individual belongs, and doctors. This research also shows that the general intention to accept preventative mHealth technology is influenced by the social influence of healthy adults. In another investigation, people used social interactions with their peers as an active information-seeking strategy to rule out potential negative effects of using or not using a certain technology. In this way, social interaction assists in lessening uncertainty by serving as a mechanism for gathering knowledge and excluding alternatives [14].

Li et al [66] evaluated a model of trusting bases along with 8 different factors in the context of initial trust in a national identity system. They found that in the setting of initial trust, social influence had a greater impact on trusting beliefs than any of the trusting bases. It is crucial because initial trust formation is particularly pertinent in information systems, where users must get past their concerns about risk and uncertainty before utilizing a technology. The closest related work to ours is the one by Scholl and Sassenberg [52], which explored whether a person’s level of identification with 2 groups, namely, (1) with the beneficiaries of app use (ie, people in their social surroundings) and (2) the source endorsing the app (ie, government officials) predicts their propensity to utilize contact tracing apps. Their results indicate that the more people identify with members of their social environment (the beneficiaries) and the government (the source), the more their app acceptance increases. We have focused on the opinion of more groups with the lens of social influence as a key driver due to the context of using the app to prevent an infection with an infectious disease and warn other members of the society in case one is sick. Therefore, we contribute to the literature by increasing the detail concerning the specific social group in question and disentangling potential relations among the influencing powers of these different groups.

## Methods

### Overview

In this section, we briefly cover the data collection, sample demographics, and the questionnaire development (see Multimedia Appendix 1 for the questionnaire).

### Data Collection and Demographics

We conducted the study with a certified panel provider in Germany (certified following the ISO 20252 norm [67]). The survey was implemented with the software LimeSurvey (version 2.72.6; LimeSurvey GmbH) [68], hosted on a university server and conducted in 2 waves. The first wave was ran in January 2021 and the second wave was ran from mid-October 2021 to mid-November 2021.

**Figure 1:**
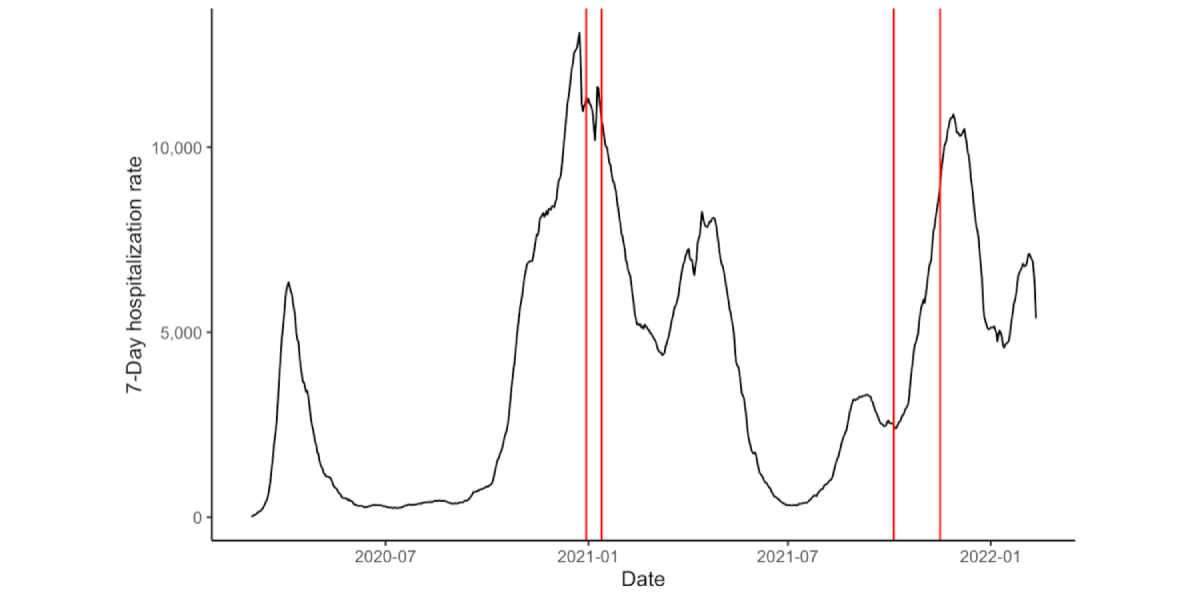
Hospitalization rate in Germany [64].

**Figure 2:**
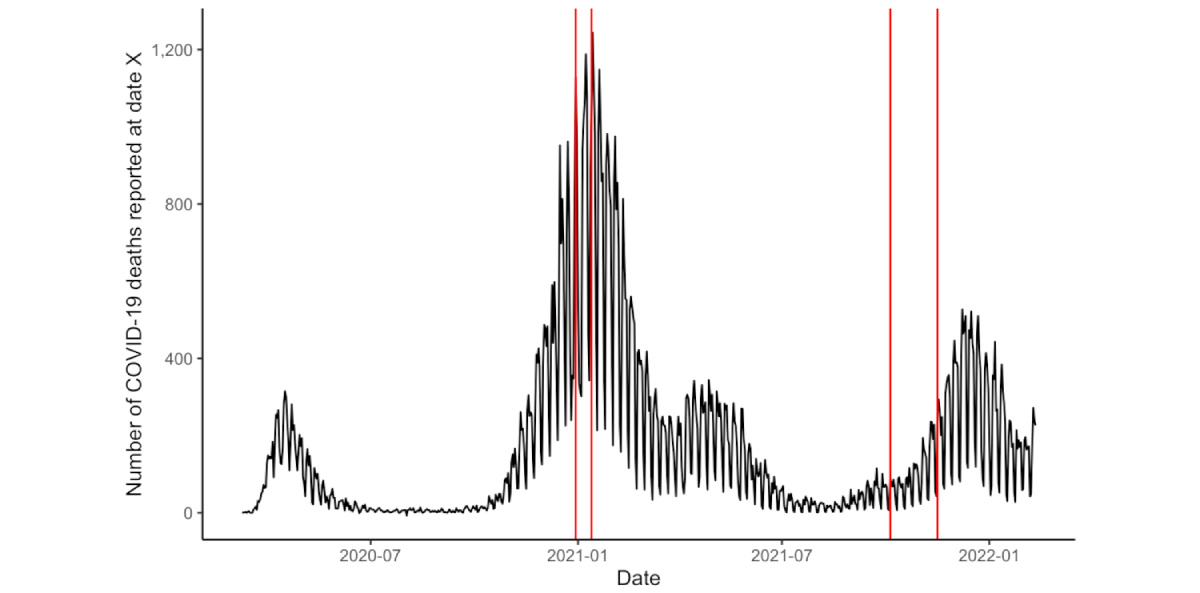
Number of SARS-CoV-2 deaths in Germany [64].

The idea behind the 2 waves was to collect data in 2 points of time with different acuteness and severeness of the pandemic (Figures 1 and 2). We chose hospitalization and death rates, as politicians in Germany decided upon disease prevention measures (eg, lockdowns) based on these 2 measures later in the course of the pandemic (initially, the incidence rate was used as the main indicator for political decisions).

In the first wave, we sampled the participants to achieve a representative sample for Germany. For that purpose, we set quotas to end up with approximately 418/833 (50.2%) females and 415/833 (49.8%) males in the sample and distribution of age following the EUROSTAT 2018 census [69]. Furthermore, we set a quota to end up with half of the sample using the CWA and the other half not using it.

In the second wave, we could only rely on the participants of the first wave. Therefore, we did not sample using hard quotas but steered participation by sending out invitations to participate in bunches. Each bunch addressed the underrepresented participants to balance the properties *use of the CWA*, *age,* and *gender*.

### Questionnaire

To assess the opinion of relevant peer groups and their influence on the participant, we asked 2 questions in a matrix, where the participant was asked about each peer group’s opinions on the app’s usage as well as how the opinion of each group influenced the participant for or against using the CWA. There was no suitable construct, thus we developed the 2 questions based on existing literature on perceived opinions [7] and influence [8] of related research. As relevant peer groups, we identified media (eg, print media, websites, television), family doctors, politicians, virologists/RKI (a German federal government agency and research institute responsible for disease control and prevention), social media, and friends/family based on discussions in the public press. The items for the peer groups’ opinions were measured with a 7-point Likert scale, ranging from “1=strongly negative” to “7=strongly positive.” The items for the peer groups’ influence were measured with a 7-point Likert scale, ranging from “1=strongly against the use of the app” to “7=strongly for the use of the app.” In addition, we gathered the demographics age, gender, education, and income of the participants.

We conducted a pretest with 12 researchers in a workshop. Each researcher answered the question independently. Afterward, we discussed the items and clarified their understanding and meaning. For perceived opinion and influence, only minor changes were made concerning the peer group names.

### Ethical Considerations

Users were informed about the purpose of the study, about the storage location of the survey data, and that they stay anonymous as long as they do not reveal their identity within the free texts. However, we used an identifier from the panel provider to link the date for each participant across the 2 waves. We did not have any further information from the panel provider linked to the identifier. Minors were not allowed to participate. This was ensured by our panel provider and an additional information text before our survey. Participants agreed that their data are used for research and consequent publications.

The user study was evaluated by the Joint Ethics Committee of the Faculty of Economics and Business of Goethe University Frankfurt and the Gutenberg School of Management and Economics of the Faculty of Law, Management and Economics of Johannes Gutenberg University Mainz. The project has been classified as “ethically acceptable.”

### Data Analysis

The data have been analyzed using SPSS version 26 (IBM, Inc.) and R (R Foundation). In the first step, descriptive statistics were used to show how users and nonusers perceived the opinions of relevant groups and their influence. In the second step, as the data were not normally distributed, 2 related samples tests (including mean, SD, minimum, maximum, number of nonmissing cases, and quartiles. Tests: Wilcoxon signed-rank, sign, McNemar) and nonparametric tests (Wilcoxon) were applied to understand how the opinions and the influence differed between users and nonusers and changed over time (from wave 1 to wave 2). And finally, using the marginal model with the generalized estimating equations, we estimated how the different groups in the participants’ social environments influenced the usage of the CWA.

## Results

### Overview

In this section, the result of the data analysis is reported. We have 2 main parts in this section: First, we briefly discuss RQ1, which is primarily a descriptive analysis of our sample. Then, second, in the data analysis part, we present the results of the remaining 3 RQs.

### Data Collection and Demographics

Our sample from the first wave consisted of 1752 participants. Following EUROSTAT 2018, participants were representatives of Germany concerning age and gender, income, and education (cf. [3,4]); 896 participants use the CWA (51.14%), whereas 856 do not (48.86%). As this is a longitudinal study with the goal to compare changes over time, we only considered the participants that took part in waves 1 and 2. This left us with 833 participants who were roughly split into 2 equally sized groups of the CWA users and nonusers (Table 1).

As we deliberately divided the sample into 2 approximately equal groups (CWA users and nonusers), we needed to ensure that the groups were not biased with respect to the demographics (Table 2). For age, we conducted a Shapiro-Wilk test for normality and found that the variable was not normally distributed (*P*<.001). Therefore, we used a Wilcoxon signed-rank sum test and found that there were no significant differences in terms of age between CWA users and nonusers (*P*=.85). We also conducted Pearson chi-square tests and found that age (*P*=.62) and gender (*P*=.09) did not reveal a statistically significant difference between users and nonusers. However, for income (*P*=.002) and education (*P*=.008), there were statistically significant differences between users and nonusers, with both of these variables being statistically significantly higher for the users compared with the nonusers. To evaluate the effect size, we additionally conducted Kendall τ test and found that the correlation between users/nonusers and their income (*P*=.01, τ=0.085) as well as education (*P*<.001, τ=0.116), respectively, was only small. Based on this result, we argue that the absolute difference does not have a substantial confounding effect on our later analysis.

**Table 1:** Participant’s use of the Corona-Warn-App over time.

Usage/wave	Wave 1 (N=833)	Wave 2 (N=833)
Users	409	427
Nonusers	424	406

**Table 2:** Demographics of participants who took part in both waves (N=833).

Demographics		Value, n (%)
**Age**		
	18-29 years	118 (14.2)
	30-39 years	149 (17.9)
	40-49 years	166 (19.9)
	50-59 years	214 (25.7)
	60 years and older	186 (22.3)
**Gender**		
	Female	418 (50.2)
	Males	415 (49.8)
	Divers	0 (0)
	Prefer not to say	0 (0)
**Net income**		
	€500-€1000^a^	76 (9.1)
	€1001-€2000	177 (21.2)
	€2001-€3000	202 (24.2)
	€3001-€4000	146 (17.5)
	More than €4000	156 (18.7)
	Prefer not to say	76 (9.1)
**Education**		
	No degree	3 (0.4)
	Secondary school	99 (11.9)
	Secondary school^b^	278 (33.4)
	A levels	184 (22.1)
	Bachelor’s degree	108 (13.0)
	Master’s degree	147 (17.6)
	Doctorate	14 (1.7)

^a^€1=US $1.08 (data as of May 20, 2023).

^b^The German education system does not allow a 1:1 translation, therefore, there are 2 different “grades” of secondary school.

### RQ1: How Do Users and Nonusers Perceive Opinions of Relevant Groups and Their Influence?

**Figure 3:**
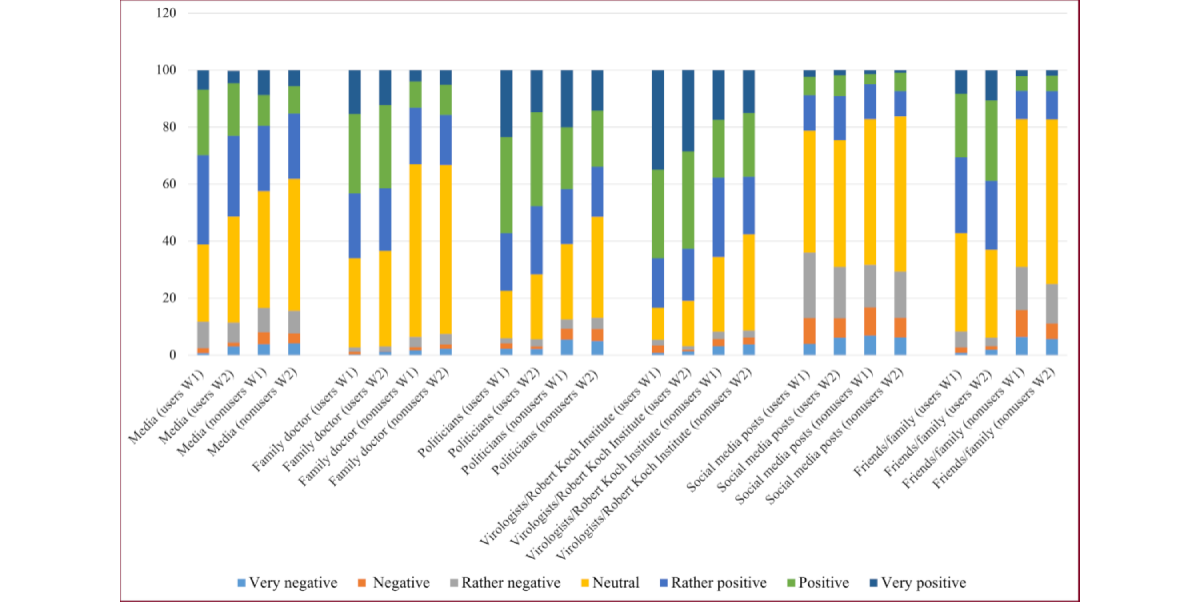
Distribution of the answers regarding the perceived opinion of different groups in participants’ social environments. W: wave.

**Figure 4:**
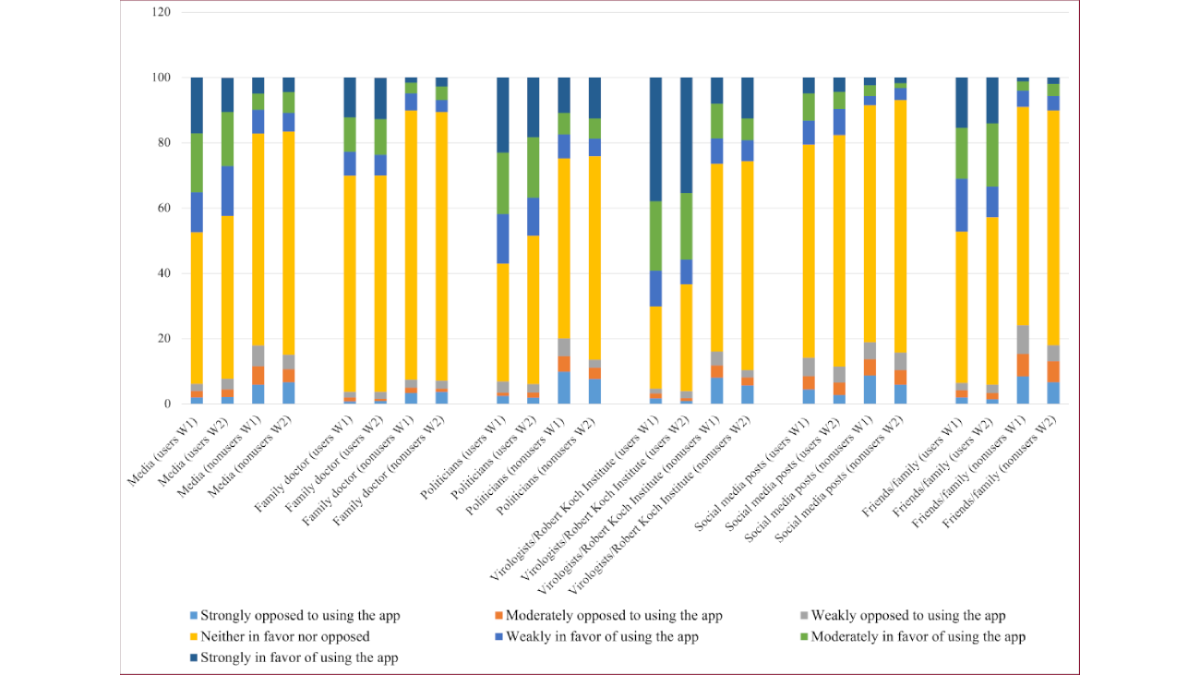
Distribution of the answers regarding the perceived influence that different groups in participants’ social environments have on using the Corona-Warn-App. W: wave.

To get an impression about the distribution of users and nonusers and investigate RQ1, we analyzed the distribution of the participants’ peer groups’ opinions and their perceived influence on the participants (Figures 3 and 4).

**Figure 5:**
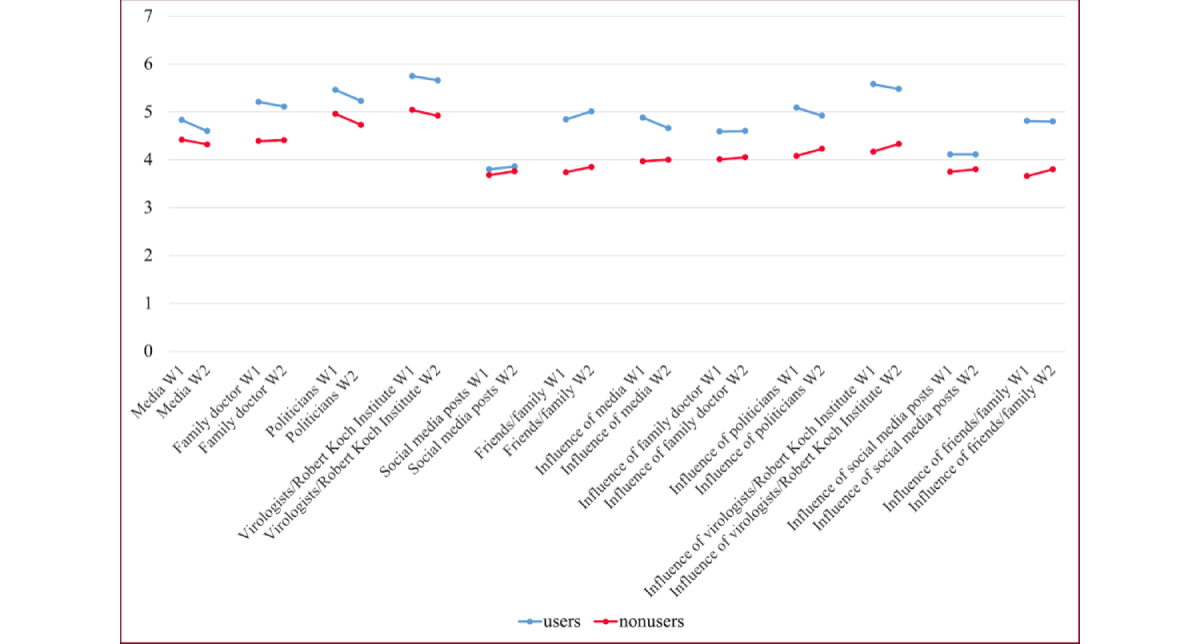
The mean of group opinion and its influence on (non)users at 2 waves. W: wave.

Figure 5 illustrates that the participants’ perception of their peer groups is in general positive, with a higher opinion from media, family doctors, politicians, and virologists/RKI. The perception of social media posts was slightly negative for both users and nonusers. Interestingly, the reported opinions from users for friends and family were way higher than the ones from nonusers; besides, the ones from nonusers were slightly negative. A similar picture was perceived when considering the influence of friends and family.

### RQ2: What Are the Differences Between Users and Nonusers?

As discussed in the previous section, CWA users seem to perceive their peer groups’ opinions more positively. Thus, we now took up RQ2 and systematically investigated the differences between users and nonusers. The visual impression from Figure 5 is supported by Mann-Whitney tests showing significant differences between users and nonusers except for the opinion of social media postings. According to the means, nonusers generally had a lower mean at both waves (Table 3).

Furthermore, we investigated the influence of gender with a Mann-Whitney *U* test. The test results indicated that gender does not present any difference in the perception of the peer groups’ opinion when it comes to the opinion of social media posts toward the CWA. The mean was higher for men than for women in both groups (user and nonuser) and in both waves (Multimedia Appendix 2).

We also investigated the influence of age on the perceived opinion and influence of the peer groups. For this purpose, we used a Kruskal-Wallis *H* test (Table 4). The result showed that the differences were significant between the different age groups for the opinions of virologists/RKI (*P*=.03) and friends/family (*P*=.04), as well as for the influence of the media (*P*<.001), family doctors (*P*=.03), politicians (*P*<.001), virologists/RKI (*P*<.001), and friends/family (*P*<.001). Although there is a tendency within these groups that the oldest group had the highest values, the means do not give a clear picture, as there was another peak for the “40-49-year” age group.

**Table 3:** Differences of perceived opinions and influence between users and nonusers

Variable	Mann-Whitney significance (*P* value)	Mean			Wave 1				Wave 2		
		User	Nonuser	Mann-Whitney significance (*P* value)		User, mean	Nonuser, mean	Mann-Whitney significance (*P* value)		User, mean	Nonuser, mean
Opinion of media	<.001	4.71	4.37	<.001		4.83	4.42	<.001		4.60	4.32
Opinion of family doctor	<.001	5.16	4.40	<.001		5.21	4.39	<.001		5.11	4.41
Opinion of politicians	<.001	5.34	4.84	<.001		5.46	4.96	<.001		5.23	4.73
Opinion of virologists/Robert Koch Institute	<.001	5.70	4.98	<.001		5.75	5.04	<.001		5.66	4.92
Opinion of social media posts	.23	3.83	3.72	.63		3.80	3.68	.23		3.86	3.76
Opinion of friends/family	<.001	4.93	3.79	<.001		4.84	3.74	<.001		5.01	3.85
The influence of media	<.001	4.77	3.98	<.001		4.88	3.97	<.001		4.66	4.00
The influence of family doctor	<.001	4.59	4.03	<.001		4.59	4.01	<.001		4.60	4.05
The influence of politicians	<.001	5.00	4.16	<.001		5.09	4.08	<.001		4.92	4.23
The influence of virologists/Robert Koch Institute	<.001	5.53	4.25	<.001		5.58	4.17	<.001		5.48	4.33
The influence of social media posts	<.001	4.11	3.77	<.001		4.11	3.75	<.001		4.11	3.80
The influence of friends/family	<.001	4.81	3.73	<.001		4.81	3.66	<.001		4.80	3.80

**Table 4:** Opinions and influence with respect to using the Corona-Warn-App for age.

Kruskal-Wallis *H* test in terms of age			Age among users, mean											z			Significance
			20-29 years		30-39 years		40-49 years		50-59 years		≥60 years						
**Groups/variable based on**																	
	Opinion of virologists/Robert Koch Institute	5.55		5.48		5.81		5.79		6.08		10.56			.03		
	Opinion of friends/family	4.78		4.62		4.92		4.79		5.13		9.69			.04		
	The influence of media	4.78		4.67		5.05		4.64		5.34		17.62			<.001		
	The influence of family doctor	4.45		4.46		4.65		4.43		5.00		10.40			.03		
	The influence of politicians	5.07		5.01		5.12		4.80		5.57		13.95			<.001		
	The influence of virologists/Robert Koch Institute	5.35		5.53		5.56		5.42		6.07		14.71			<.001		
	The influence of friends/family	4.59		4.72		4.73		4.69		5.36		13.43			<.001		

### RQ3: How Do the Opinions and the Influence Change Over Time (From Wave 1 to Wave 2) Driven by Infection Rates (Decreased From Wave 1 to Wave 2)?

We also investigated the changes in the perceived opinion and the influence of peer groups over time. Table 5 shows that the differences among users in the first wave and second wave were minimal. However, after applying the Wilcoxon test, we found significant differences:

- Users’ perceived opinion about the media group was significantly lower in the second wave. This holds true for all participants (*P*=.001) and for the users (*P*=.001), but the decrease for nonusers was lower (*P*=.15), and not statistically significant.
- Users’ perceived opinion of politicians was significantly lower for all participants (*P*<.001) as well as for the user (*P*=.004) and nonuser (*P*=.003) subgroups in wave 2.

- Users’ perceived opinion of friends/family with respect to using the CWA had significantly increased for all participants (*P*>.001) as well as for the user (*P*=.003) and nonuser (*P*=.02) subgroups.
- The perceived influence of media toward using the CWA significantly decreased among users (*P*=.002), meaning the influence was weaker but still toward using the CWA.
- The perceived influence of virologists/RKI toward using the CWA significantly increased among nonusers (*P*=.01) toward using the CWA.
- The perceived influence of friends/family toward using the CWA significantly increased among nonusers (*P*=.02) toward using the CWA.

**Table 5:** Differences in opinion and influence between the 2 points in time (waves 1 and 2).

Variable	Users				Nonusers				Wilcoxon significance (*P* value) of all participants
	Wilcoxon significance (*P* value)	Wave 1, mean	Wave 2, mean	Wilcoxon significance (*P* value)		Wave 1, mean	Wave 2, mean		
Opinion of media	.001	4.83	4.60	.15		4.42	4.32	.001	
Opinion of family doctor	.18	5.21	5.11	.24		4.39	4.41	.86	
Opinion of politicians	.004	5.46	5.23	.003		4.96	4.73	<.001	
Opinion of virologists/Robert Koch Institute	.23	5.75	5.66	.15		5.04	4.92	.06	
Opinion of social media posts	.18	3.80	3.86	.40		3.68	3.76	.13	
Opinion of friends/family	.003	4.84	5.01	.02		3.74	3.85	<.001	
The influence of media	.002	4.88	4.66	.52		3.97	4.00	.11	
The influence of family doctor	.66	4.59	4.60	.12		4.01	4.05	.53	
The influence of politicians	.08	5.09	4.92	.20		4.08	4.23	.90	
The influence of virologists/Robert Koch Institute	.08	5.58	5.48	.01		4.17	4.33	.47	
The influence of social media posts	.94	4.11	4.11	.59		3.75	3.80	.74	
The influence of friends/family	.80	4.81	4.80	.02		3.66	3.80	.09	

### RQ4: How Does the Influence of the Relevant Groups Influence the Usage of the CWA?

We used a marginal model with generalized estimating equations to investigate the effect of (non)users’ social environment on the usage of the CWA (Table 6). As can be seen, among social environment variables, the influence of politicians (*P*=.04), virologists/RKI (*P*<.001), and friends/family (*P*<.001) was significant and had changed the usage of the CWA. The other variables were insignificant (media: *P*=.13; family doctor: *P*=.80; social media: *P*=.07; and time: *P*=.26), and their change did not affect the independent variable. The influence of virologists/RKI had the most increasing effect (increasing the odds of using the CWA). Increasing 1 unit of influence of the virologists/RKI variable and keeping the other variables constant increased the odds of using the CWA by 44%. The only decreasing effect (decreasing the odds of using the CWA) was related to the influence of politicians variable. Increasing 1 unit of the influence of politicians variable and keeping the other variables constant decreased the odds of using the CWA by 10%.

We modeled time as a single variable to represent the influence of the different waves to have a simple model and reduce complexity. Modeling it as an interaction term with each of the other independent variables would have resulted in not only a more complex model, but also one with certain overlaps (each variable with its interaction of time), which are hard to interpret.

To investigate how the opinion and the influence of peer groups are correlated, we conducted a Pearson correlation. The Pearson correlation shows that the opinion of the social environment and its influence on using the CWA are related but not strongly correlated (Table 7).

**Table 6:** Marginal model with generalized estimating equations for the effects of the influence of the social environment on using the app.

Parameter		B	Hypothesis test		Exp(B)
			Wald chi-square	Significance (*P* value)	
(Intercept)		–2.641	107.323	<.001	0.071
Influence of media (print media, websites, film and television)		0.085	2.328	.13	1.088
Influence of family doctor		–0.012	0.062	.80	0.988
Influence of politicians		–0.098	4.073	.04	0.907
Influence of virologists/Robert Koch Institute		0.363	47.024	<.001	1.437
Influence of social media posts		–0.086	3.261	.07	0.917
Influence of friends/family		0.308	46.023	<.001	1.361
Time		0.069	1.291	.26	1.072
**Goodness of fit**					
	Quasi likelihood under independence model criterion^a^	NA^a^	NA	NA	1931.927
	Corrected quasi likelihood under independence model criterion	NA	NA	NA	1938.039

^a^NA: not applicable.

**Table 7:** The Pearson correlation between opinion toward the social environment and its influence on using the Corona-Warn-App.

Correlation			User		Nonuser
**Opinion of media and its influence on using the app**					
	Pearson correlation (*r*)	0.397		0.219	
	Significance (*P* value)	<.001		<.001	
**Opinion of family doctor and its influence on using the app**					
	Pearson correlation (*r*)	0.395		0.337	
	Significance (*P* value)	<.001		<.001	
**Opinion of politicians and its influence on using the app**					
	Pearson correlation (*r*)	0.383		0.156	
	Significance (*P* value)	<.001		<.001	
**Opinion of virologists/Robert Koch Institute and its influence on using the app**					
	Pearson correlation (*r*)	0.496		0.252	
	Significance (*P* value)	<.001		<.001	
**Opinion of social media posts and its influence on using the app**					
	Pearson correlation (*r*)	0.346		0.324	
	Significance (*P* value)	<.001		<.001	
**Opinion of friends/family and its influence on using the app**					
	Pearson correlation (*r*)	0.434		0.377	
	Significance (*P* value)	<.001		<.001	

## Discussion

Following the structure of the previous section, we discuss the RQs one by one.

### RQ1: How Do Users and Nonusers Perceive Opinions of Relevant Groups and Their Influence?

It is surprising that the perception of participants with respect to their peer groups is in general positive. Given that half of the participants were not using the CWA, we assumed that the perception of the nonusers’ peer groups would be on the lower side of the Likert scale. However, that is only the case for family and friends and social media posts. Social media posts are only perceived to be slightly negative, but the opinion expressed there is perceived lower than from all other groups. This might be related to the ongoing discussions about the Network Enforcement Act (German: Netzwerkdurchsetzungsgesetz, NetzDG), which tries to combat fake news, harassment, and misinformation in social media.

### RQ2: What Are the Differences Between Users and Nonusers?

Not surprisingly, users perceive their peer groups’ opinions more positively than nonusers. Gender does not seem to have an influence. However, with increasing age the tendency increased to perceive the peer groups’ opinion more positively. However, there was a peak for the “40-49-year” age group for all peer groups. The “40-49-year” age group overlaps to a large degree with the so-called Gen X, but we could not find any indication that SARS-CoV-2 or technology was perceived differently by this group in Germany.

### RQ3: How Do the Opinions and the Influence Change Over Time Driven by Infection Rates?

Hospitalization rates and number of deaths were significantly lower during wave 2 compared with wave 1. The perceived opinion of media and politicians has significantly decreased from wave 1 to wave 2. This fits with the observation that many politicians were (wrongly) blaming the app to be not so useful because it does not send information to the public health departments or blaming data protection for hindering the effectiveness of the app.

The perceived opinion of friends and family as well as their perceived influence toward using the CWA has increased. This might be related to the perception that many public health departments in Germany were overloaded and the official fight against SARS-CoV-2 was given up due to shortage of staff.

### RQ4: How Does the Opinion of the Relevant Groups Influence the Usage of the CWA?

The influence of virologists/RKI has the most increasing effect. This could be backed up by a huge presence of the RKI in the media and their decisive role in changing the rules during the pandemic. The only decreasing effect we found was the influence of politicians, which could be explained by the participants getting tired of politicians contradicting each other, feathering their own nest by promoting companies selling masks and other medical equipment to fight the pandemic, and a number of seemingly uncoordinated decisions between the different states and the federation.

### Principal Findings

Our results indicate that participants’ perception of their peer groups is in general positive, with a higher opinion from media, family doctors, politicians, and virologists/RKI and a lower, only slightly negative, opinion from social media posts. Users perceived their peer group’s opinion higher than nonusers. A similar pattern can be observed when considering the peer groups’ influence instead of the opinion. The perceived opinion of media and politicians has significantly decreased from wave 1 to wave 2. The perceived opinion of friends and family as well as their perceived influence toward using the CWA has increased. The influence of virologists/RKI has the most increasing effect. The only decreasing effect we found was the influence of politicians.

### Limitation

Our study has several limitations. First, our measurement of the opinion and influence of the participants’ peer groups was self-reported. On the one hand, participants might report not only wrong values, but also misinterpret their own perception. On the other hand, this is supported by our evaluation that the participants’ perception is more important than the actual opinion of the peer groups. As a consequence, it is unclear whether lower values stem from a lower perception or a lower opinion (ie, for the report of nonusers).

Furthermore, we can only evaluate correlations but not causality. Therefore, we do not know whether the users’ perception of their peers’ opinion is higher, because they are using the app. In contrast to nonusers, they might be able to identify wrong statements within their peer group and disregard them.

In addition, we only differentiated between users and nonusers. There might be different levels of activity when using the app (ie, participants might just look at infection rates or the personal risk or they could share their own infection).

The separation of groups is not very strict, that is, participants could read statements of the RKI or from other peer groups via social media. However, there is most likely a different perception between those groups; therefore, we had included social media as its own group in the survey.

Our study only had participants located in Germany using the CWA. While the study could not easily be transferred to other countries, as all countries have different contact tracing apps, there might still be cultural influences in the perception of and interaction with the named peer groups. Thus, it could be interesting to have similar investigations in other countries or cultures in the future.

### Comparison With Prior Work

To the best of our knowledge, different entities of the social environment of users and nonusers and their influence on the usage of contact tracing apps have not been investigated yet. Only 1 study by Scholl and Sassenberg [52] is related to ours because it investigated the social environment of contract tracing users by measuring a person’s level of identification with the beneficiaries of the contact tracing app (ie, people in their social surroundings) to predict their willingness to use contact tracing apps. Thus, this study is only partially related as it covers only one of the groups we also asked for in our study, namely, friends and family. The authors found that the closer other people in individuals’ social environments are, the more likely they are to use contact tracing apps. This is in line with our finding that a positive opinion and influence of friends and family positively influence the use of the CWA. We contribute to the literature by widening the analysis to different peer groups in the social environment such as doctors or politicians. In addition, our variable social influence is conceptually different from the identification variable in the study by Scholl and Sassenberg [52].

In addition, Oldeweme et al [14] investigated the influence of transparency, social influence, trust in the government, and initial trust in a COVID-19 tracing app on the process of adopting the app. Their results showed that the transparency dimensions of disclosure and accuracy, in addition to social influence, trust in government, and initial trust, positively influenced the adoption process. They agree on the definition of social influence as the “degree to which an individual perceives that important others believe he or she should use the new system” [5], but they did not investigate which groups were more important than others.

Social influence not only directly influences the adoption of the CWA, but might also influence other important antecedents of the adoption. We have already mentioned that peer groups most likely have an influence on the knowledge of the app [4], which itself influences the privacy concerns, and thus the adoption of the app [3]. Additionally, the perception of the perceived disease threat has been shown to influence the adoption of the app when applying the TAM [70], the Health Belief Model [71], and the Protection Motivation Theory [55]. However, peer groups might also influence the perceived disease threat. Kaspar [55] found that the intention for using a contact tracing app increased when trust in other people’s social distancing behavior decreased. Although other people might be not considered as a peer group, it clearly shows that the perceived behavior of other people influences the adoption of the app. However, Kaspar [55] did not further investigate differences between specific groups’ influence on the adoption. Kostka and Habich-Sobiegalla [13] investigated the public perception toward COVID-19 tracing apps in Germany (and China and the United States) and examined variables such as conspiracy belief (not significant), belief in a second wave (significant), or trust in the state (partially significant). However, they did not investigate the influence of peer groups, although connections have been demonstrated between the COVID-19 pandemic and the 5G conspiracy theory and the spread of misinformation in social networks [72].

Alam et al [73] made use of the Health Belief Model to investigate the public attitude toward vaccinations against COVID-19. They found that, among other factors, “health motivation” was an important factor for the willingness to get vaccinated. Part of this construct is the recommendation of friends, relatives, and the participants’ physician. However, they also did not further investigate the influence of the different groups.

### Conclusions and Future Work

Opinions of peer groups play an important role when it comes to the adoption of the CWA. Naturally, not all groups have the same importance. Our results show that the influence of virologists/RKI and family and friends contributed to the adoption of the CWA the most, while politicians only had a slightly negative influence on citizens to use the CWA. Our results indicate that it is crucial to accompany the introduction of such a contact tracing app with an appropriate media campaign with easily understandable technical explanations and the clear approval of political decision makers to support its adoption among a large group of citizens in a given country.

Although the pandemic is considered by many to be overcome, these considerations are still important to make, to create a more resilient society in the future. It is important to investigate not only the adoption of contact tracing apps, but also the adoption of data donation apps. Although the CWA has a feature using which users can report their infections, it would also be beneficial if data could be collected to learn more about the specific disease and how it spreads. For that purpose, not only privacy and privacy concerns should be investigated, but also the influence of peer groups, as they can play a decisive role in the adoption of apps. Besides contact tracing and data donation apps, apps could be used to nudge the users into specific behaviors, such as physical distancing [74], which again would rely on the users’ intention to adopt the app(s).

One natural idea of a future work is to extend our study to other health apps such as those mentioned earlier. One could go even further and investigate health apps such as fitness tracking apps or diet diary apps in general. However, it is also important to scientifically connect the different areas and study the interdependencies of knowledge, perceived disease threat, the opinion and influence of peer groups, and the adoption of the CWA. However, media and misinformation or fake news can influence people’s opinion about the CWA. Therefore, besides a solid education and online/computer literacy, it is important to understand the effects of peer groups to be able to plan and implement governmental information campaigns accordingly.

## Appendix

Multimedia Appendix 1 Questionnaire used in the survey.

Multimedia Appendix 2 Mann-Whitney *U* test for gender.

## Abbreviations

CWA: Corona-Warn-App
mHealth: mobile health
RKI: Robert Koch Institute
RQ: research question
TAM: Technology Acceptance Model
