# Supplementary material for: The Impact of Individuals’ Social Environments on Contact Tracing App Use: Survey Study
Source: JMIR Hum Factors. 2023 May 31;10:e45825. doi: 10.2196/45825 (PMC10234420; doi:10.2196/45825)
Supplement: Multimedia Appendix 1 [file humanfactors_v10i1e45825_app1.docx]

**Multimedia Appendix 1**

**Demographics**

We asked for the following demographics, answer options are listed in brackets:

- Age^y^
- Gender (female, male, divers)^n^
- Education (no degree, secondary school, secondary school (>5 GCSE), A levels, bachelor, master, doctorate)
- Household income (in e: 0.5k-1k, 1k-2k, 2k-3k, 3k-4k, >4k)^n^
- Corona-Warn-App user (yes/no)

^n^Prefer not to say option ^y^ in years

**Opinion of Peers**

1. How do you perceive the opinion of the following groups on the use of the Corona-Warn-App? ^^[[1]](#footnote-1)^^

- Media (print media, websites, film and television)
- Family doctor
- Politicians
- Virologists / Robert Koch Institute
- Social media posts
- Friends / Family

1. How did the opinion of each group influence you?^^[[2]](#footnote-2)^^

- Media (print media, websites, film and television)
- Family doctor
- Politicians
- Virologists / Robert Koch Institute
- Social media posts
- Friends / Family

1. Scale: 1 Very negative, 2 Negative, 3 Rather negative, 4 Neutral, 5 Rather positive, 6 Positive, 7 Very positive [↑](#footnote-ref-1)
2. Scale: 1 Strongly opposed to using the app, 2 Moderately opposed to using the app, 3 Weakly opposed to using the app, 4 Neither in favor nor opposed to using the app, 5 Weakly in favor of using the app, 6 Moderately in favor of using the app, 7 Strongly in favor of using the app [↑](#footnote-ref-2)
