# Supplementary material for: The Impact of Individuals’ Social Environments on Contact Tracing App Use: Survey Study
Source: JMIR Hum Factors. 2023 May 31;10:e45825. doi: 10.2196/45825 (PMC10234420; doi:10.2196/45825)
Supplement: Multimedia Appendix 2 [file humanfactors_v10i1e45825_app2.docx]

| Mann-Whitney U test for gender | User | | | | Non-user | | | |
| --- | --- | --- | --- | --- | --- | --- | --- | --- |
|  | W1 | | W2 | | W1 | | W2 | |
|  | U | sig | U | sig | U | sig | U | sig |
|  |  |  |  |  |  |  |  |  |
| Media | 20672.500 | .851 | 22329.000 | .746 | 22304.000 | .910 | 20085.500 | .696 |
| Family doctor | 19520.500 | .236 | 21016.500 | .163 | 21829.000 | .580 | 20393.500 | .904 |
| Politicians | 19681.000 | .296 | 21983.500 | .547 | 21097.500 | .276 | 20381.000 | .903 |
| Virologists / Robert Koch Institute | 20192.500 | .543 | 22495.000 | .851 | 22277.000 | .894 | 20437.500 | .942 |
| Social media posts | 17641.000 | .004 | 20159.500 | .034 | 19435.000 | .010 | 18290.500 | .038 |
| Friends / family | 20663.500 | .845 | 22280.500 | .718 | 21836.000 | .604 | 20166.000 | .738 |
| The influence of Media | 19463.000 | .205 | 20556.000 | .067 | 21368.500 | .319 | 19784.500 | .449 |
| The influence of Family doctor | 19404.000 | .139 | 22526.000 | .852 | 21739.500 | .401 | 20454.500 | .934 |
| The influence of Politicians | 20350.000 | .640 | 21728.000 | .407 | 21586.000 | .457 | 20117.500 | .695 |
| The influence of Virologists / Robert Koch Institute | 20034.000 | .455 | 20972.000 | .149 | 21354.500 | .338 | 20369.000 | .881 |
| The influence of social media posts | 18966.000 | .058 | 22387.500 | .741 | 21008.500 | .148 | 18370.500 | .013 |
| The influence of Friends / family | 20511.500 | .737 | 22391.500 | .777 | 22411.500 | .978 | 18879.500 | .079 |
